# Ponatinib in the treatment of patients with chronic myeloid leukemia and increased cardiovascular risk: A review of management strategies

**DOI:** 10.1016/j.htct.2024.04.124

**Published:** 2024-08-17

**Authors:** Tomasz Sacha, Katarzyna Krawczyk

**Affiliations:** Jagiellonian University Medical College, Kopernika, Kraków, Poland

**Keywords:** Ponatinib, Chronic myeloid leukemia, Tyrosine kinase inhibitor, Cardiovascular risk, Management strategy

## Abstract

The introduction of tyrosine kinase inhibitors has revolutionized the treatment of chronic myeloid leukemia vastly improving the prognosis and clinical outcome of most patients. It was estimated that approximately 40–50 % of patients treated with imatinib will require treatment with a second-generation or third-generation tyrosine kinase inhibitor to achieve an optimal response. The treatment duration, increased patient survival, and aging of the population receiving tyrosine kinase inhibitors raise concerns as to long-term toxicities, such as an elevated cardiovascular risk and a higher rate of comorbidities. Ponatinib is a highly potent third-generation tyrosine kinase inhibitor that was shown to be effective in patients with a wide range of ABL mutations, including T315I. The use of ponatinib is associated with significant vascular toxicity, including peripheral arterial occlusive disease, ischemic heart disease, cerebrovascular accidents, and venous thromboembolism. This review discusses the vascular toxicity of ponatinib and presents a comprehensive panel of tests for the evaluation of patients requiring ponatinib therapy. Moreover, the management of patients with cardiovascular risk factors who receive ponatinib is discussed. Finally, the strategy for establishing the optimal dose of ponatinib in patients with chronic myeloid leukemia is described.

## Introduction

Chronic myeloid leukemia (CML) is a clonal myeloproliferative disorder arising from abnormal hematopoietic stem cells harboring a reciprocal chromosomal translocation between the long arms of chromosomes 9 and 22. The translocation results in the formation of a short chromosome 22, called the Philadelphia chromosome, and the generation of the *BCR::ABL1* oncogene in almost all CML cases.^1^^,^^2^ The fusion *BCR::ABL1* gene is transcribed into a hybrid mRNA and translated into the BCR::ABL1 protein with constitutive and abnormally high tyrosine kinase activity.

The discovery of molecular mechanisms underlying the development and progression of CML has driven the design and development of tyrosine kinase inhibitors (TKIs). These drugs have revolutionized the treatment of CML, leading to a dramatic improvement in the prognosis and clinical outcome of most patients. The first-generation TKI, imatinib, was introduced in 2000. Since then, the annual mortality rate in patients with CML has decreased from 10 to 20 % to 1–2 %,^3^ thus providing enough time for different types of complications to develop. It was estimated that approximately 40–50 % of patients treated with imatinib will require treatment with a second-generation or third-generation TKI to achieve an optimal response.^4^^,^^5^ As second-generation TKIs are more potent inhibitors of BCR::ABL1 tyrosine kinase than imatinib, they have all been tested as first-line therapy and were shown to induce faster cytogenetic and molecular responses in a higher number of patients compared with the first-generation drug.^6^, ^7^, ^8^, ^9^, ^10^, ^11^, ^12^ Hence, there is considerable interest in exploring the potential use of second-generation TKIs in a broader population of patients. However, long-term toxicities were reported, such as elevated cardiovascular risk, and these concerns may limit the use of TKIs, particularly in specific patient subsets.^11^^,^^13^ Moreover, extended treatment duration and patient survival result in the aging of the population receiving TKIs. Consequently, these patients have higher rates of comorbidities, such as atherosclerosis and its complications, lipid and glucose metabolism disorders, congestive heart failure, or hypertension.

Ponatinib (Takeda/Incyte - Iclusig) is a highly potent third-generation TKI, which can inhibit a wide range of tyrosine kinases and was shown to effectively target all known single-resistance ABL kinase mutations.^14^^,^^15^ The drug may be required in adult patients with a chronic, accelerated, or blast phase of CML with resistance or intolerance to previous treatment with a first-generation or second-generation TKI and who are ineligible for subsequent treatment with imatinib. Importantly, as the drug is specifically designed with a carbon-carbon triple bond, it can target the T315I point mutation within the kinase domain of BCR::ABL1.^16^^,^^17^ The T315I mutation is observed in approximately 20 % of CML patients who have developed resistance to other TKIs, such as imatinib, nilotinib, dasatinib, and bosutinib. So far, ponatinib has been the only approved TKI to demonstrate clinically significant activity against this mutation. Moreover, so far, only the T315M mutation in the ATP-binding site has been identified to confer resistance to ponatinib.^18^

## Vascular toxicity of ponatinib

The use of ponatinib can lead to significant vascular toxicity, causing various diseases such as peripheral arterial occlusive disease, ischemic heart disease, cerebrovascular accidents, and venous thromboembolism. The Phase 2 PACE trial (Ponatinib Ph+ ALL and CML Evaluation; ClinicalTrials.gov identifier, NCT01207440) revealed a correlation between ponatinib administration and serious arterial occlusive events (AOEs). In vitro studies also revealed that ponatinib treatment increases endothelial cell dysfunction and apoptosis, leading to a higher rate of adverse vascular events. Ponatinib-associated adverse vascular events are likely related to endothelial inflammation, dysfunction, and apoptosis.^19^ The incubation of cultured human aortic endothelial cells with ponatinib induced an elevation in the phosphorylation of the nuclear factor kappa B (NF-κB)/p65, as well as an increase in NF-κB activity, inflammatory gene expression, cell permeability, and cell apoptosis. Moreover, ponatinib reduced the expression of ERK5-responsive genes, including Krüppel-like factor 2/4 and endothelial nitric oxide synthase. Ponatinib was also found to enhance ERK5 SUMOylation, which hampers its transcriptional activity, transforming endothelial cells into an inflammatory phenotype and disrupting vascular homeostasis.^20^ Additionally, the drug demonstrated the ability to induce apoptosis, reduce migration, and inhibit tube formation of human umbilical vein endothelial cells, along with exerting a negative impact on the function of endothelial progenitor cells.^21^

In vitro studies further revealed that ponatinib induced apoptosis in human coronary artery endothelial cells in a dose-dependent manner. Moreover, ponatinib was found to inhibit the proliferation of human umbilical vein endothelial cells and HMEC-1 cells. It also inhibited fetal bovine serum-induced phosphorylation of the vascular endothelial growth factor (VEGF) receptor KDR, insulin receptors, and the MER receptor tyrosine kinase, which are involved in vascular homeostasis, angiogenesis, and vessel protection. The findings indicate that the antiangiogenic activity of ponatinib may be attributed to its ability to inhibit VEGF signaling at the receptor level and downstream pathways.^22^ Moreover, ponatinib stimulates the production of vasoconstricting prostanoids^19^ and increases the mRNA expression of coagulation factors associated with both the contact activation and tissue factor pathways. Consistent with these effects, ponatinib leads to elevated plasma levels of factor VII.^23^

## The incidence and risk factors of arterial occlusive events in patients treated with ponatinib

The incidence of AOEs related to ponatinib administration in patients enrolled in the PACE trial was recently reassessed by an independent adjudication committee consisting of five academic experts (three cardiologists, one vascular medicine specialist, and one vascular neurologist).^24^ The trial included patients diagnosed with CML or Philadelphia chromosome–positive acute lymphoblastic leukemia who had shown resistance or intolerance to dasatinib or nilotinib, or had a detectable *BCR::ABL1* T315I mutation, regardless of prior TKI treatment.^25^ All participants were administered a starting ponatinib dose of 45 mg/d. In patients achieving at least a major cytogenetic response, it was possible to reduce the dose to 30 mg/d or 15 mg/d. However, if the patient did not achieve at least a major cytogenetic response, the dose was typically reduced to 30 mg/d, unless the investigator decided otherwise based on the benefit-to-risk assessment considering the patient's disease characteristics, *BCR::ABL1* mutation status, and cardiovascular risk.

The endpoints in this study were adjudicated on the basis of established definitions included in the 2014 American College of Cardiology/American Heart Association guidelines,^26^ as well as definitions for cardiovascular and stroke outcomes developed by the Standardized Data Collection for Cardiovascular Trials Initiative and the US Food and Drug Administration (FDA).^27^^,^^28^ All potential AOEs identified during the pharmacovigilance search were evaluated using the predefined definitions outlined in the study charter.^24^ These definitions encompassed various clinical conditions such as myocardial infarction, heart failure if attributed to an AOE (including coronary artery disease, arterial hypertension, cardiomyopathy, or myocardial infarction), hospitalization for unstable angina, stroke, and other cerebrovascular events, as well as peripheral vascular disease. Adjudicated AOEs were determined based on the events that met the specified criteria for each endpoint, including the requirements such as revascularization, changes in cardiac biomarkers, and diagnostic evidence supported by imaging techniques like computed tomography scans or magnetic resonance imaging.

The relative risk of AOEs was assessed based on the baseline risk category in patients belonging to the safety population, where data for all baseline risk categories were available. These risk categories encompassed commonly acknowledged cardiovascular risk factors, including arterial hypertension, hypercholesterolemia, diabetes mellitus, obesity, as well as a history of heart disease (non-ischemic or ischemic). The baseline history of non-ischemic cardiovascular disease (CVD) was reported for 43 % of patients, and a history of ischemic CVD, for 23 %. The frequency of adjudicated AOEs was lower than of non-adjudicated AOEs. In patients with chronic-phase CML (CP-CML), the rates of adjudicated AOEs (57/270; 21 %) were also lower than the rates of non-adjudicated AOEs (84/270; 31 %). Among CP-CML patients with adjudicated AOEs, 95 % (54/57) had serious AOEs. Peripheral arterial occlusive disease was the only adjudicated AOE that occurred with low prevalence (16/449; 4 %). The most common non-adjudicated AOEs determined through adjudication were angina pectoris, non-cardiac chest pain, and chest pain. These events were often documented as symptoms or preliminary diagnoses with a low severity level and without any changes associated with medication or hospitalization.

Over time, the study showed a decrease in the incidence of newly occurring AOEs when adjusted for exposure. The median time for the first adjudicated AOE to occur was 14.1 months (range: 0.1–49.5 months). Most patients (46 %) continued taking ponatinib after experiencing an AOE without any changes to the drug dosage. Additionally, 35 % of patients had their doses reduced and/or interrupted following the event. A small proportion of patients (9 %) decided to discontinue ponatinib specifically due to an adjudicated AOE. The primary risk factors observed in patients who experienced an adjudicated AOE were arterial hypertension and hypercholesterolemia, which were also among the most prevalent baseline risk factors. Patients with adjudicated AOEs also more often used concurrent antihypertensive medications, platelet aggregation inhibitors, and antidiabetic agents when compared with those without AOEs.

The rate of adjudicated AOEs based on the number of baseline risk factors (including arterial hypertension, hypercholesterolemia, obesity, diabetes mellitus, non-ischemic cardiac disease, and ischemic disease) was as follows: 13 % (24/189) among patients with 1–2 risk factors and 29 % (52/180) among patients with 3 or more risk factors. Of the 80 patients who had no risk factors at baseline, only two (3 %) experienced an AOE. These findings are consistent with previous studies.^29^^,^^30^ Of the 11 patients who experienced AOEs leading to death, nine had a documented history of cardiovascular events and/or cardiovascular risk factors at baseline. It is noteworthy that the long-term survival of patients with adjudicated AOEs was comparable to the survival of patients who did not experience an AOE, which suggests that it is the progression of CML itself rather than the occurrence of AOEs that plays a significant role in determining the outcome of these patients.

## Cardiovascular risk assessment

In general, the overall cardiovascular risk should be considered when deciding about the intensity of therapy or about the pharmacological treatment for hypercholesterolemia and arterial hypertension.^31^ Cardiovascular risk assessment becomes particularly important in patients requiring treatment with a second-generation or third-generation TKI or with a STAMP (specifically targeting the ABL myristoyl pocket) inhibitor. The assessment of overall cardiovascular risk also facilitates patient education.^32^ In this context, the calculation of vascular age is particularly useful.^33^

Cardiovascular risk is defined as the likelihood of CVD or death due to CVD within a specific timeframe. Total cardiovascular risk refers to the risk estimated based on all factors present in an individual. This is a theoretical concept because it is practically impossible to assess all factors. Therefore, in practice, the concept of overall cardiovascular risk is used, which refers to the risk estimated based on selected main factors.

Available risk scores, which incorporate data on various conventional cardiovascular risk factors, typically calculate the individual's risk over ten years. For example, the Systemic Coronary Risk Estimation (SCORE)^33^, ^34^, ^35^, ^36^ assesses the ten-year risk of fatal CVD in high-risk regions of Europe (such as Poland, Czech Republic, Slovakia, and Hungary, among other countries) based on sex, age, systolic blood pressure, total cholesterol, and smoking status. This score is recommended for estimating total cardiovascular risk in over 40-year-old adults, unless they are automatically classified as ‘high risk’ or ‘very high risk’ due to documented CVD, diabetes mellitus (for over 40-year-old individuals), kidney disease, or significantly elevated single risk factors such as cholesterol or blood pressure. A risk level of 10 % or higher is categorized as very high; 5 % or higher, as high; 1–5 %, as intermediate; and less than 1 %, as low. In June 2021, two new scores, SCORE2 and SCORE2-OP, were published to assess the individual's ten-year risk of fatal and nonfatal CVD (myocardial infarction, stroke) in apparently healthy individuals with untreated or stable risk factors over several years. SCORE2 is designed for patients aged between 40 and 69 years, while SCORE2-OP is used for risk assessment in patients aged between 70 and 89 years (Table 1).^34^^,^^35^^,^^37^ The Heart score calculator utilizing SCORE2 and SCORE2-OP is available online (https://www.heartscore.org/en_GB/).

**Table 1:** The SCORE2 and SCORE2-OP risk categories.

Risk category	A 10-year risk of fatal cardiovascular disease
Very high risk	≥10 %
High risk	5 %–9 %
Intermediate risk	1 %–4 %
Low risk	<1 %

The Framingham score, which is a sex-specific algorithm created in 1998, is also designed to assess the ten-year cardiovascular risk of an individual. Over time, it has undergone modifications and updates, specifically incorporating dyslipidemia, age, hypertension treatment, smoking, and total cholesterol levels. Importantly, diabetes, which was included in the earlier version, was excluded because it was deemed to be equivalent to coronary heart disease.^38^

The initial Framingham score for assessing overall cardiovascular risk was shown to predict the likelihood of experiencing a cardiovascular event in patients on nilotinib therapy.^39^ This implies that patients at risk of a cardiovascular event during treatment with TKIs could be identified at an early stage, which highlighted the importance of proactive monitoring as well as managing comorbidities and cardiovascular risk factors in all patients (see https://www.mdcalc.com/calc/38/framingham-risk-score-hard-coronary-heart-disease). In our previous study, we assessed the vascular endothelial function in patients with CML who received different TKIs. Our findings indicated that the impaired endothelial function in conduit arteries and microcirculation did not align with SCORE/SCORE2/SCORE2-OP estimation. Specifically, the group of patients classified as having low or moderate risk according to SCORE/SCORE2/SCORE2-OP did not show significant differences compared with patients classified as having high or very high risk. We concluded that endothelial dysfunction observed in CML patients treated with TKIs is not associated with cardiovascular risk as evaluated by these risk scores.^71^ This suggests that the SCORE assessment and the functional evaluation of the endothelium should be considered separately when assessing CVD risk in these patients. Incorporating the examination of endothelial function could enhance the prediction of cardiovascular risk in CML patients as well as help design optimal treatment plans.

Despite numerous studies analyzing risk factors for cardiovascular events during TKI treatment for CML, an accurate prediction of these events remains challenging and an effective prediction tool is yet to be developed. The occurrence of cardiovascular events associated with TKI treatment is significantly affected by the presence of comorbidities in individual patients, and the incidence of these events is higher in patients who are at increased cardiovascular risk. Therefore, it is crucial to recognize and address cardiovascular risk factors before and during the administration of ponatinib and other TKIs.^18^^,^^40^^,^^41^

## Cardiovascular risk assessment before ponatinib therapy

According to guidelines that address cardiovascular toxicity in oncology, patients at risk of cardiovascular events should be closely monitored before and during treatment. It is important to implement strategies aimed at reducing cardiovascular risk factors throughout the duration of treatment and then after the completion of treatment.^42^, ^43^, ^44^

At baseline, the assessment is needed to enable the early identification of patients who are at high risk of cardiovascular complications. The criteria for the ‘high-risk’ category may differ slightly depending on the guidelines.^42^, ^43^, ^44^, ^45^ The general criteria are presented in Table 2.

**Table 2:** High-risk patients according to a definition included in the guidelines on cardio-oncology.^45^, ^72^

• patients with previous cardiovascular diseases or their risk factors: ○ older than 60, 65 or 75 years old, ○ female sex and genetic factors ○ with 2 or more established risk factors for cardiovascular disease (diabetes mellitus, dyslipidemia, chronic renal insufficiency, hypertension, obesity, smoking history), ○ elevated heart markers (NT-proBNP, troponin) ○ compromised cardiac function (borderline low LVEF 50–55%, history of MI, moderate or severe valvular heart disease) • those exposed to high-dose anthracycline therapy and radiotherapy (involving the heart in the field) • and those undergoing co-therapy with other cardiotoxic modalities

There is currently no established panel of diagnostic tests for a comprehensive cardiovascular risk assessment at baseline in patients with cancer. The available guidelines recommend clinical evaluation, electrocardiography (ECG), left ventricular ejection fraction (LVEF) assessment by echocardiography, and baseline blood pressure measurement. Additionally, in high-risk patients, cardiac markers should be assessed.^42^, ^43^, ^44^ The Cardio-Oncological Evaluation Model developed by a panel of experts includes a clinical consultation (with blood pressure measurement), ECG, blood glucose, lipid profile, glomerular filtration rate, cardiovascular global risk assessment (based on the guidelines), as well as echocardiographic assessment of LVEF and global longitudinal strain.^45^ These guidelines provide a comprehensive approach to the early detection and monitoring of potential cardiac complications in individuals undergoing all types of cancer treatment. As for the ponatinib therapy, various studies suggested similar methods, with an additional testing of the adverse event profile of TKIs. This includes risk factor assessment based on medical history, ECG, and echocardiographic LVEF measurement, and, additionally, evaluation of the ankle-brachial index (ABI) and cardiac ankle vascular index.^46^ Other proposed approaches further expand the diagnostic panel to include blood pressure measurement, basic metabolic panel, fasting glucose and glycated hemoglobin A1c, as well as fasting lipid panel.^47^

It was reported that AOEs occurred during ponatinib therapy even in patients without previously detected ischemic risk factors. Therefore, it is necessary to implement more complex vascular diagnostic tests. One of the proposed approaches is the use of peripheral vascular ultrasound to detect atherosclerotic plaques and to measure the intima-media thickness of the supra-aortic and limb vessels before and during ponatinib therapy. If this method is not feasible, the measurement of the ABI serves as an immediate tool for assessing the risk of peripheral arterial disease (PAD).^47^^,^^48^ By calculating the ratio between the systolic blood pressure in the ankle and arm, the ABI provides a sensitive and specific measure for diagnosing PAD. Moreover, it is a reliable predictor of mortality and adverse cardiovascular events, independent of traditional cardiovascular risk factors. Normal ABI values range from 1.0 to 1.4. Values lower than 0.9 are considered diagnostic of PAD, while values below 0.5 indicate severe PAD.^47^ Doppler ultrasound of the lower limb veins is recommended only in patients with a history of venous thrombosis.^48^

### Medical history

According to the World Health Organization (WHO), the most important behavioral risk factors of heart disease and stroke in under 50-year-olds are unhealthy diet, physical inactivity, tobacco use, and harmful use of alcohol.^49^ The effects of these risk factors may manifest as elevated blood pressure, increased blood glucose and lipid levels, as well as overweight and obesity^49^ Therefore, a medical history for cardiovascular risk should include the following:

1) a family history of cardiovascular disorders, particularly premature CVD (i.e., in patients aged <50 years old);
2) presence of arterial hypertension, diabetes mellitus, hypercholesterolemia;
3) cardiovascular symptoms: chest pain, shortness of breath on exertion and rest, arrhythmias, bleeding or petechiae, limb pain on movement, symptoms of thrombosis;
4) respiratory symptoms: cough or shortness of breath (which may indicate pleural effusion), pulmonary hypertension, or interstitial lung disease;
5) previous TKI treatment;
6) concomitant medications with possible interactions with TKIs; the patient should be asked specifically about drugs prolonging the QTc interval on ECG (see https://crediblemeds.org/pdftemp/pdf/CombinedList.pdf or https://crediblemeds.org) as well as P450 (CYP3A) inhibitors or inducers (see https://www.fda.gov/drugs/drug-interactions-labeling/drug-development-and-drug-interactions-table-substrates-inhibitors-and-inducers);
7) lifestyle and physical activity, smoking, alcohol consumption, eating habits, sedentary habits;
8) other comorbidities.

The assessment of the Charlson comorbidity index can also be considered (Table 3) (see https://www.mdcalc.com/calc/3917/charlson-comorbidity-index-cci).

**Table 3:** Charlson comorbidity index.

Comorbidity	Score
Previous myocardial infarction	1
Congestive heart failure	1
Peripheral vascular disease	1
Cerebrovascular disease	1
Chronic lung disease	1
Rheumatic disease	1
Peptic ulcer disease	1
Mild liver disease	1
Diabetes mellitus	1
Cerebrovascular (hemiplegia) event	2
Moderate to severe kidney disease	2
Diabetes with chronic complications	2
Cancer without metastasis	2
Leukemia	2
Lymphoma	2
Moderate or severe liver disease	3
Metastatic solid tumor	6
AIDS	6

### Physical examination

A thorough physical examination is recommended, with a special emphasis on treatment-related symptoms (Table 4).

**Table 4:** Typical symptoms during treatment with tyrosine-kinase inhibitors (TKIs).^41^

Symptoms	TKI
• peripheral edema • periorbital edema • skin redness/rash	Imatinib
• jaundice • hepatomegaly or splenomegaly • abdominal tenderness (suggesting liver or pancreatic disease) • pallor, coldness, absence of pulse in the limbs • carotid murmur • cardiac arrhythmia • orthostatic edema	Nilotinib
• additional breath sounds over the lungs • abnormalities on cardiac examination • bleeding or petechiae • orthostatic edema and other symptoms of fluid retention • cardiac arrhythmia • other symptoms suggestive of heart failure	Dasatinib
• abdominal pain • jaundice • other symptoms of liver or pancreatic disease	Bosutinib
• headaches or other symptoms suggesting high blood pressure • abdominal tenderness (suggesting liver or pancreatic disease) • pallor, coldness, absence of pulse in the limbs • carotid murmur • cardiac arrhythmia • orthostatic edema	Ponatinib

Finally, the risk category should be determined as proposed in Table 5. Additional cardiotoxicity factors should also be considered, such as heart failure (with either preserved or reduced LVEF), asymptomatic LVEF (<50 % or high natriuretic peptide levels), hypertensive heart disease with left ventricular hypertrophy, all types of cardiomyopathy, cardiac sarcoidosis with myocardial involvement, and significant cardiac arrhythmias (e.g., ventricular tachyarrhythmias or atrial fibrillation).

**Table 5:** Risk categories based on European cardiovascular disease and SCORE2/SCORE2-OP risk assessment.

Risk category	Patients with any of the following:
Very high risk Corresponds to SCORE2/SCORE2-OP ≥10 %	1. Documented CVD on clinical examination: • previous myocardial infarction • acute coronary syndrome • coronary revascularization • coronary artery bypass grafting or other arterial revascularization procedures • stroke and transient ischemic attack • peripheral arterial disease 2. Documented CVD on imaging: significant plaque on coronary angiography or carotid ultrasound 3. Severe CKD (GFR <30 mL/min/1.73 m^2^) 4. Diabetes mellitus with end-organ damage (e.g., proteinuria) 5. Major risk factor present (e.g., smoking, hypertension, or dyslipidemia)
High risk Corresponds to SCORE2/SCORE2-OP 5–9 %	1. Significant elevation in the levels of a single risk factor: • Blood pressure ≥180/110 mmHg • Cholesterol >8 mmol/L (>310 mg/dL) 2. Moderate CKD (GFR 30–59 mL/min/1.73 m^2^) 3. Diabetes mellitus
Intermediate risk	SCORE2/SCORE2-OP 1–4 %
Low risk	SCORE2/SCORE2-OP <1 %

Abbreviations: CKD: chronic kidney disease; CVD: cardiovascular disease; GFR: glomerular filtration rate.

The proposed model of preliminary and follow-up examinations for cardiotoxicity in patients considered for ponatinib treatment is presented in Table 6.

**Table 6:** The proposed schedule of cardiovascular toxicity assessment in patients on ponatinib treatment.

Procedure to be done initially	During therapy
Medical history with the reporting of any cardiovascular symptoms or events	Every 3 months
Patient education on risk factor prevention (smoking cessation, healthy diet, physical exercise)	Every visit
Physical examination	Every 3 months
Blood Pressure and pulse assessment	Daily home monitoring
Cardiovascular risk assessment (SCORE)	Annually
Blood tests: complete blood count with differential, creatinine clearance (GFR), fasting glucose, glycated hemoglobin (Hb1Ac), lipids	Every 3 months
Blood tests: cardiac markers BNP, NT-proBNP, troponin I	Every 6 months in ≥intermediate cardiovascular risk
Blood tests: lipase, amylase	Every 3 months
Electrocardiography	Every 12 months in low cardiovascular risk and every 6 months in all other risk categories
Echocardiography: LVEF and GLS (if available)	Every 12 months in low cardiovascular risk and every 6 months in all other risk categories
ABI and/or supra-aortic and limb peripheral ultrasound assessment	Every 12 months in intermediate risk and every 6 months in ≥high cardiovascular risk
Specialist consultation: diabetologist, cardiologist, vascular medicine specialist, or cardio-oncologist (if available) (in ≥intermediate cardiovascular risk)	As per physician's recommendation

Abbreviations: ABI, ankle-brachial index; GFR, glomerular filtration rate; GLS, global longitudinal strain; LVEF, left ventricular ejection fraction;.

## Management of patients with cardiovascular risk factors

### Prevention of adverse cardiovascular events

Early identification of high-risk patients allows healthcare providers to implement proactive measures to monitor cardiovascular health, thus potentially minimizing the impact of complications and optimizing patient outcomes. Therefore, the second part of the preliminary assessment should focus on the implementation of primary or secondary preventive measures. According to experts in cardio-oncology, this involves the active management of modifiable cardiovascular risk factors and CVD, as well as promoting regular exercise and healthy dietary habits.^45^ If baseline cardiotoxicity risk is determined to be high, the use of cardioprotective medication as prophylaxis is recommended.^42^ A prevention model known as the ABCDE approach (Table 7) has been proposed to mitigate cardiovascular risk in patients with CML undergoing treatment with TKIs.^50^ This approach aims to address the potential cardiovascular risks associated with TKI therapy and provide a framework for reducing such risks. In the case of ponatinib, implementing the ABCDE approach may help balance the drug-related risk and optimize the cardiovascular health of patients. By following this approach, healthcare professionals can proactively monitor and manage cardiovascular risk factors, whereby the impact of CVD in TKI-treated patients can be minimized.

**Table 7:** The ABCDE approach to primary prevention of cardiovascular disease. Modified from^50^.

A	B	C	D	E
**Awareness -** understanding cardiovascular risk factors as well as signs and symptoms of CVD **ABI -** assessment of peripheral arteries **Acetylsalicylic acid -** after weighing the pros and cons	**Blood pressure control -** home blood pressure monitoring is recommended; hypertension should preferably be treated with ACEIs/ARBs, dp-CCBs, or β-blockers (ndp-CCB should be avoided.	**Cigarette cessation; cholesterol lowering** – statins, diet modification, physical exercise	**Diet** - controlling serving portion, high fiber, low trans fats, cholesterol, and salts, eating low-fat protein sources **Diabetes management**	**Exercise** - >30 min of moderate exercise 4 times/wk = >120 min/wk **Electrocardiography** **Echocardiography**

Abbreviations: ABI, ankle-brachial index; ACEI, angiotensin-converting enzyme inhibitor; ARB, angiotensin receptor blocker; CVD, cardiovascular disease; dp-CCB, dihydropyridine calcium channel blocker; ndp-CCB, non-dihydropyridine calcium channel blocker.

Unequivocal data on the effectiveness of antithrombotic prophylaxis in high-risk patients undergoing ponatinib treatment are lacking. It remains unclear whether primary prevention with antithrombotic agents provides a significant benefit in this setting.^47^ As the approach to thromboprophylaxis depends on an individual patient's risk, the decision should be based on risk assessment and after carefully weighing the risks and benefits of antithrombotic treatment. Patients should be managed according to current guidelines and clinical practice standards and in consultation with a specialist physician. Regular monitoring for thrombotic complications is necessary.

As part of risk factor management, patients should be advised on lifestyle modifications, including a healthy diet, regular exercise, smoking cessation, optimal blood pressure as well as lipid and glucose control.

### Patient monitoring during ponatinib therapy

There are currently no clear recommendations for the monitoring of patients during ponatinib therapy. Based on Phase 1 and 2 trials, the median time to the onset of cerebrovascular events is 526 days from the initiation of therapy, and to the onset of limb events, 478 days.^51^^,^^52^ These findings make it clear that patients receiving ponatinib should be monitored with regular checkups.

Experts differ in their recommendations on the approach to monitoring. Cavecchia et al.^47^ suggested that Doppler ultrasound of the supra-aortic and limb arteries is performed every year in patients without vascular disease or increased intima-media thickness at baseline, and at least every six months in patients who develop atherosclerotic plaques during ponatinib treatment. In a review article, Manouchehri et al.^50^ recommended that the initial assessment is repeated one month after the initiation of treatment and then every 3–6 months. A panel of experts in cardio-oncology suggested regular evaluation for cardiotoxicity similar to that performed at baseline, with home blood pressure monitoring repeated every three months during the first year of ponatinib treatment, and then every six months. ^45^ Screening for PAD using the ABI and Doppler ultrasound of the supra-aortic and lower limb arteries should be repeated every six months in patients at high and very high cardiovascular risk, or annually in patients at intermediate risk.^45^

After an initial assessment and analysis of risk factors, it is necessary to determine the risk category. In low-risk patients, a non-pharmacological strategy is recommended, including lifestyle interventions and exercise pre-habilitation. This approach appears to have a beneficial effect on cardiovascular risk factors. In patients at high cardiovascular risk, all cardiovascular risk factors should be adequately addressed, which requires a strict pharmacological control of blood pressure, dyslipidemia, and diabetes. In patients with a significantly elevated cardiovascular risk, it is crucial to assess whether the benefits of treatment outweigh potential risks as well as to clarify whether baseline cardiovascular risk will affect the choice of TKI. This should be done in consultation with a diabetologist, cardiologist, vascular medicine specialist, or cardio-oncologist. This approach is aimed at optimizing both primary and secondary prevention measures.

The proposed approach to monitoring, with a list of measures and suggested timing, is shown in Table 7. However, as the access to specialized diagnostic tests may vary across the different healthcare settings, a tailored approach should be adopted, utilizing the available diagnostic tools to the best extent possible. Ultimately, the goal is to achieve a balance between the individual patient's risk profile, the benefits of comprehensive monitoring, and the capabilities of a given healthcare setting. A close collaboration between the treating oncologist, cardiologist, and other relevant healthcare professionals is crucial for making informed decisions about the frequency and type of tests performed as part of patient monitoring during treatment.

### The initial dose of ponatinib

According to the 2020 recommendations by the European LeukemiaNet (ELN), patients with chronic-phase CML are eligible for ponatinib treatment if they are resistant to or do not tolerate two or more TKIs. Additionally, ponatinib should be considered in those patients in whom another TKI, including imatinib, is not suitable. The optimal therapy should be selected based on the patient's age, comorbidities, and cardiovascular risk factors, as well as clinical response to previous TKI treatment. According to the ELN guidelines, a reduced starting dose of 30 mg/d or 15 mg/d is recommended for patients with a lower level of resistance or intolerance, particularly in the presence of cardiovascular risk factors. The presence of the T315I mutation, compound mutations, disease progression to the accelerated or blast phase, or Philadelphia chromosome-positive acute lymphoblastic leukemia is an indication for a starting dose of 45 mg/d.

**Figure 1:**
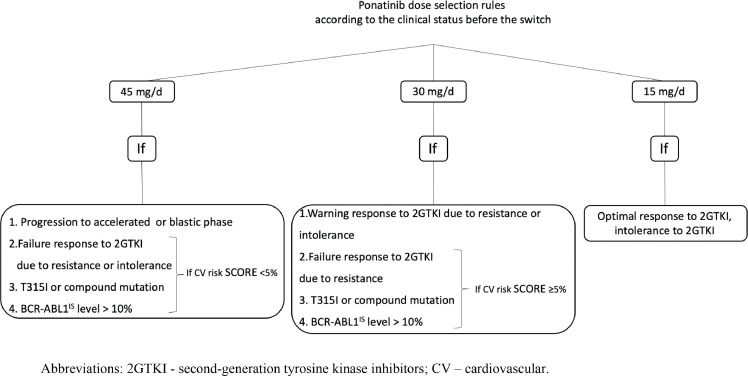
Recommendations on initial dose selection of ponatinib in patients with chronic myeloid leukemia (CML).

Abbreviations: 2GTKI, second-generation tyrosine kinase inhibitors; CV, cardiovascular.

Real-life studies provide insights into the risk-benefit profile of ponatinib dosing in patients with CP-CML. Based on these data an algorithm for the treatment of this patient population was developed.^53^ The authors suggested that prior to ponatinib treatment, the reason for switching to ponatinib should be identified. In the case of treatment failure, particularly with the BCR::ABL1 level higher than 10 %, or in the presence of T315I or compound mutations, the recommended starting dose of ponatinib is 45 mg/d. If the TKI is switched to ponatinib because of a warning response, the starting dose should be 30 mg/d. In patients with intolerance to two or more TKIs, it is crucial to assess response to previous treatments. Patients who have not achieved an optimal response should be started on ponatinib at the same dose as patients with resistance, while those with an optimal response can start treatment at 15 mg/d. However, an exception should be made for patients with a high or very high cardiovascular risk based on the European Society of Cardiology guidelines (Table 1). Patients who remain in the chronic phase should receive a starting dose of either 30 mg/d or 15 mg/d, regardless of the reason for switching to ponatinib (including patients with treatment failure). A slightly modified algorithm for selecting the initial dose of ponatinib in CML patients is presented in Figure 1.

In a review article, Molica et al.^54^ discussed the optimal use of ponatinib in patients with CP-CML. Based on findings from clinical trials and real-life studies in patients with CP-CML, they concluded that even low starting doses (30 mg/d or 15 mg/d) can induce or maintain major molecular response or deep molecular response with a potential reduction in the incidence of cardiovascular events. The authors recommended a starting ponatinib dose of 30 mg/d or 15 mg/d to reduce potential drug-related risks. Such dosage should also be considered in patients who have already achieved a major molecular response and developed significant intolerance to previously used TKIs. Moreover, a special consideration should be given to patients who have experienced a thrombotic event with other previous TKIs. Low-dose ponatinib should be considered in these patients only if all other TKIs have already been used.^54^

Finally, a German expert consensus panel recommended initiating treatment with 30 mg/d in patients with CP-CML without ABL kinase domain mutations, resistant to only one TKI, with a good response status, intolerant to TKI despite a good response, and with increased cardiovascular risk.^55^

### The strategy for ponatinib dose reduction

The initial dose of 45 mg/d was established in the Phase 2 PACE trial ^24^ subsequently approved by the FDA and European Medicines Agency. However, pharmacodynamic studies revealed that the dose of 30 mg/d or higher resulted in the trough plasma concentrations of ponatinib exceeding 40 nM, thus effectively inhibiting all tested BCR::ABL1 mutants in preclinical studies. ^25^ Additionally, a dose of 15 mg/d led to a minimum 50 % reduction in CRKL phosphorylation (a marker of BCR::ABL1 activity) in 32 of 34 patients (94 %), including eight of ten patients (80 %) with the T315I mutation.

The association between ponatinib dose and the occurrence and severity of adverse vascular events was suggested in a report^56^ combining data from a Phase 1 dose-escalation study,^57^ Phase 2 PACE trial,^52^ and Phase 3 EPIC (Evaluation of Ponatinib versus Imatinib in Chronic Myeloid Leukemia) trial.^58^ For every reduction of 15 mg in the daily ponatinib dose, the risk of AOEs was reduced by 33 %.^56^ The Phase 2 OPTIC trial (Optimizing Ponatinib Treatment in CP-CML) aims at assessing the effectiveness and safety of ponatinib in patients with CP-CML who are resistant to at least two TKIs or who have the T315I mutation.^60^ The trial utilizes a novel dose-adjustment strategy based on treatment response, with the goal of optimizing the efficacy and enhancing the safety profile of ponatinib in patients with highly resistant CP-CML. The primary analysis of OPTIC revealed a favorable risk-to-benefit ratio for ponatinib when employing the response-based dosing approach, starting with a dose of 45 mg/d and then reducing it to 15 mg/d upon achieving a BCR::ABL1^IS^ level of ≤1 % assessed along with the International Scale (IS).

In the OPTIC trial, the incidence of serious adverse events and AOEs among patients receiving a dose of 45 mg/d was lower compared with the PACE trial, with rates of 31.2 % versus 63.4 % and 5.6 % versus 20.2 %, respectively.^59^ The exposure-adjusted treatment-emergent AOE rates were 5.6 %, 3.6 %, and 2.1 % for the 45-mg, 30-mg, and 15-mg cohorts, respectively. Grade 3 to 5 treatment-emergent AOEs were reported in 13 patients (4.6 %; five patients in the 45-mg cohort; five patients in the 30-mg cohort, and three patients in the 15-mg cohort). ^60^ Benefits were also seen with the starting doses of 30 mg/d and 15 mg/d in patients without the T315I mutation and in those with less resistant disease, indicating that molecular characteristics may be useful in further refining risk-adapted therapy strategies.^60^

These findings suggest that even with a prompt dose reduction to 15 mg/d after achieving a BCR::ABL1 level of ≤1 %, the efficacy of ponatinib is maintained and the drug considerably reduces the frequency and severity of AOEs. Real-life data on the safety and tolerability of lower initial doses of ponatinib support the results of clinical trials, indicating that for patients who are intolerant or exhibit low resistance to second-generation TKIs, initiating treatment with a lower ponatinib dose may be an optimal strategy. This approach has the potential to reduce the occurrence and severity of treatment-related AOEs while maintaining clinical response.^61^, ^62^, ^63^, ^64^, ^65^, ^66^, ^67^, ^68^, ^69^

The post hoc analysis of OPTIC demonstrated clinical benefits across all the three dosing regimens, regardless of the baseline BCR::ABL1 level and the presence of the T315I mutation. The primary endpoint, which involved achieving a reduction in BCR::ABL1 levels to less than 1 % at 12 months, was attained by 51 % of patients receiving a dose of 45 mg/d followed by 15 mg/d, 32 % of patients receiving a dose of 30 mg/d followed by 15 mg/d, and 33 % of patients receiving a dose of 15 mg/d. Among the subgroup of patients with the T315I mutation, the primary endpoint was achieved by 60 % of patients receiving the dose of 45 mg/d followed by 15 mg/d, 25 % of patients receiving a dose of 30 mg/d followed by 15 mg/d, and 11 % of patients receiving a dose of 15 mg/d. Regardless of the T315I mutation status, most patients maintained their response after dose reduction to 15 mg/d once they achieved a BCR::ABL1^IS^ level of ≤1 %. Patients with the T315I mutation at baseline were more susceptible to experiencing a loss of response following dose reduction; however, dose re-escalation resulted in the recovery of response in 60 % of cases. Among patients with T315I mutations, the dosing regimen of 45 mg/d to 15 mg/d showed superior progression-free survival compared with the other treatment arms. Conversely, for patients without T315I mutations, all three doses exhibited robust progression-free and overall survival outcomes.^70^

## Conclusions

Remarkable advancements in prognosis and clinical outcomes have been observed in the majority of patients undergoing treatment with tyrosine kinase inhibitors (TKIs), thereby extending treatment duration and enhancing patient survival. The aging demographic within the TKI-treated population contributes to elevated comorbidity rates, necessitating careful consideration in the strategic planning of optimal therapeutic interventions. Notably, the evaluation of CVD risk factors assumes heightened significance, especially in instances where second or third generation TKIs are deemed necessary. The incorporation of cardio-oncological support, coupled with the judicious administration of an appropriate ponatinib dosage, stands as a pivotal measure in significantly mitigating the risk of cardiovascular side effects. It is important to emphasize that the benefits of effective treatment with TKIs may outweigh the potential risk of cardiovascular complications as well as risks associated with mortality linked to disease progression. This is particularly important in patients who may have limited alternative treatment options, including patients with the BCR::ABL1 T315I mutation.

## Conflicts of interest

Tomasz Sacha received honoraria and speakers’ fees from Roche, Novartis, Bristol-Myers Squibb, Pfizer, Angelini Pharma, and GSK. Katarzyna Krawczyk received honoraria from Novartis, Pfizer, Angelini Pharma, Amgen, and Abbvie.
